# How Behavioral Constraints May Determine Optimal Sensory Representations

**DOI:** 10.1371/journal.pbio.0040387

**Published:** 2006-11-28

**Authors:** Emilio Salinas

**Affiliations:** Department of Neurobiology and Anatomy, Wake Forest University School of Medicine, Winston-Salem, North Carolina, United States of America; Columbia University, United States of America

## Abstract

The sensory-triggered activity of a neuron is typically characterized in terms of a tuning curve, which describes the neuron's average response as a function of a parameter that characterizes a physical stimulus. What determines the shapes of tuning curves in a neuronal population? Previous theoretical studies and related experiments suggest that many response characteristics of sensory neurons are optimal for encoding stimulus-related information. This notion, however, does not explain the two general types of tuning profiles that are commonly observed: unimodal and monotonic. Here I quantify the efficacy of a set of tuning curves according to the possible downstream motor responses that can be constructed from them. Curves that are optimal in this sense may have monotonic or nonmonotonic profiles, where the proportion of monotonic curves and the optimal tuning-curve width depend on the general properties of the target downstream functions. This dependence explains intriguing features of visual cells that are sensitive to binocular disparity and of neurons tuned to echo delay in bats. The numerical results suggest that optimal sensory tuning curves are shaped not only by stimulus statistics and signal-to-noise properties but also according to their impact on downstream neural circuits and, ultimately, on behavior.

## Abstract 2

A quantitative theoretical approach demonstrates how optimal sensory tuning curves are shaped not only by stimulus statistics and signal-to-noise properties, but also according to their impact on downstream neural circuits and behavior.

## Introduction

Sensory neurons respond to physical stimuli, and this relationship is often quantified by plotting their evoked activity—for instance, the mean firing rate—as a function of a relevant stimulus parameter. The resulting response functions or tuning curves have been the subject of much theoretical work, particularly relating to vision. In trying to understand such tuning curves, the emphasis has been on information maximization, the main idea being that sensory neurons should represent the sensory world as accurately and efficiently as possible [1–3]. This principled approach, known as the efficient coding hypothesis, has been extremely successful at predicting the receptive field properties of neurons in early visual [4–7] and auditory [8,9] areas, and it is consistent with numerous experimental observations [10–13].

However, information maximization is not enough. Such a principle cannot completely account for the response characteristics of cortical neurons, particularly beyond early sensory areas, because it does not consider how the encoded information will be used, if at all. It would not make sense for sensory neurons to pack a lot of information into parts of feature space that are of little relevance to the animal. A recent study [14] investigating auditory responses in grasshoppers illustrates this. Primary auditory receptors in grasshoppers do not respond equally well to different types of environmental sounds. Instead, the stimulus ensemble that maximizes their information rate consists of short segments of grasshopper songs that mark the transitions between song syllables [14]. Thus, such early receptor neurons seem to be highly specialized for describing a rather small set of sounds that are relevant for a specific behavior, namely, discriminating grasshopper songs [15].

This raises an interesting question: does an animal's behavior influence the shapes of its sensory tuning curves? If so, what features would be most sensitive to behavioral constraints? There are, in fact, two motivations for addressing this problem: first, the limitations just discussed of the efficient coding principle; second, the ubiquity of monotonic tuning curves, which I see as a theoretical mystery. Tuning curves come in two main flavors, single-peaked and monotonic (increasing or decreasing). Bell-shaped curves with a single peak are the textbook example of tuning functions. They are indeed quite common [16–20], and many modeling studies have investigated the coding properties of arrays of such unimodal curves subject to some form of noise [21–25]. Monotonic dependencies on stimulus parameters, however, have also been amply documented, not only in the somatosensory system [26–28] but also in other modalities [29–31]. Monotonic tuning curves have received little attention from theorists. No analysis has been reported from the standpoint of efficient coding, and it is not clear whether they present any advantage regarding other criteria, such as learning [32]. To complicate matters further, some neuronal populations show mixtures of monotonic and peaked curves [33–35].

Why is there such a range of tuning curve shapes? And, in particular, what promotes the development of monotonic profiles? To investigate more closely whether behavioral factors play a role in this problem, here I evaluate the responses of a neuronal population not only in relation to their sensory inputs but also in terms of the range of outputs that they are capable of generating. The sensory tuning curves are seen as a set of basis functions from which other functions of the stimulus parameters can be easily constructed [36,37]. These other functions represent motor activity or actions that are generated in response to a stimulus. The idea is that if something can be said about the statistics of the downstream motor activity, then we should be able to say something about the sensory tuning curves that are optimal for driving such activity.

## Results

### Tuning Curves as Basis Functions

**Figure 1:**
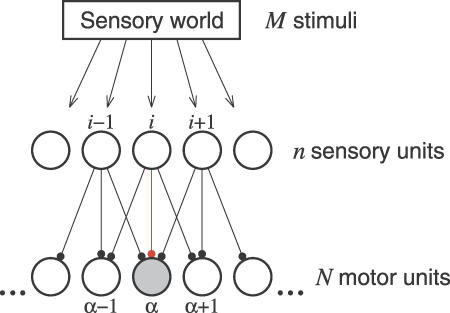
Schematic of the Model There are *n* sensory or basis neurons that respond to *M* stimuli and drive *N* motor neurons downstream. The firing rate of motor neuron *α* (shown filled) when stimulus *k* is presented is equal to *R*_α*k*_ =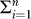 *w*_α*i*_ *r_ik_*, where *r_ik_* is the firing rate of sensory neuron *i,* and *w_αi_* is the connection (shown in red) from sensory neuron *i* to downstream neuron *α*. For each motor neuron *α,* the driven response *R_αk_* should approximate as closely as possible a desired response *F_αk_*.

To begin, the problem needs to be defined mathematically. The situation can be described using some of the tools of classic function approximation [38,39] and is schematized in Figure 1: *n* basis neurons respond to *M* stimuli or conditions and drive *N* additional downstream neurons whose output should approximate a set of desired functions **F**. The basis neurons represent sensory neurons in whose tuning curves we are interested, and the downstream units represent motor neurons that contribute to generating actions. The key quantity to study is the matrix **r**, where *r_ik_* is the firing rate of basis neuron *i* evoked by stimulus *k*. These basis responses may have intrinsic variability (noise), so their mean values are denoted as 〈*r_ik_*〉, where the brackets indicate an average over multiple presentations of the same stimulus. Because the second index parameterizes stimulus values, the tuning curve of cell *i* is simply 〈*r_ik_*〉 plotted as a function of *k*. As mentioned above, the rationale of this approach is that although the motor responses **F** may be largely unknown in reality, if they have some regularity or statistical structure, this should partially determine the optimal shapes of the sensory tuning curves 〈**r**〉. For the moment, however, pretend that the repertoire of motor responses **F** that should be elicited by the stimuli is fully known.

To proceed, a mechanism is needed for the sensory neurons to communicate with the motor neurons. The simplest assumption is that the downstream motor units are driven through weighted sums. Thus, the response of downstream unit *α* to stimulus *k* is *R_ak_* = 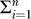 *w*_α*i*_ *r_ik_*, where *w*_α*i*_ represents the synaptic connection from sensory neuron *i* to downstream neuron *α* (Figure 1). In matrix notation, this is **R** = **wr**. In this simple model, the shapes of the tuning curves become important when there are more downstream neurons than basis neurons (*n* < *N*) and when there is noise, so both conditions are assumed to be true.

Next, recall that the job of downstream unit *α* is to produce the target motor response *F→*_α_ (where *F→*_α_ is row *α* of **F**). Therefore, what is needed is for the driven responses, **R** = **wr**, to approximate as closely as possible the desired ones, **F**. Crucially, however, different sets of tuning curves 〈**r**〉 will vary in their capacity to generate the target downstream responses. This capacity is quantified using an error measure denoted as *E_B_*. When *E_B_* is 0, the sensory (basis) neurons are most accurate and the driven responses are equal to the desired ones; when *E_B_* is 1, the driven activity has little or no resemblance to the desired activity and the error is maximal. The derivation of *E_B_* is presented in the Methods section. What is important, however, is to understand its dependencies, which are as follows: *E_B_* = *E_B_*(〈**r**〉, **σ**, {*s_k_*}, **Φ**). First, the error depends on the sensory tuning curves 〈**r**〉 and on their noise, **σ**. Second, note that there is no dependence on the synaptic weights. This is because *E_B_* is constructed assuming that, for each 〈**r**〉, the best possible synaptic weights are always used. Third, *E_B_* depends on how often each stimulus is shown; that is, on the set of coefficients {*s_k_*}, where *s_k_* is the probability that stimulus *k* is presented. Finally, *E_B_* does not depend directly on the actual motor responses **F**. Instead, the key independent quantity is their correlation matrix **Φ**, which captures their overall statistical structure. Its components are

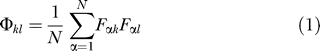
In essence, **Φ** represents an average over all the downstream motor responses that the basis neurons have to approximate. This average corresponds to drawing the *F_αk_* values from given distributions, or equivalently, to choosing multiple functions *F→*_α_ from a given class (see below).

In summary, given the noise of the neurons (**σ**), the statistics of the stimuli ({*s_k_*}), and the statistics of the downstream responses (**Φ**), the error *E_B_* can be calculated for any set of sensory tuning curves 〈**r**〉.

### What Determines the Optimal Tuning Curves?

So far, what I have done is set up the problem and developed a quantity that measures the effectiveness of the sensory tuning curves as building blocks for constructing the desired motor responses. Recall, however, that the goal is to find the best tuning curves. In the present formalism, this is the same as asking what tuning curves 〈**r**〉 minimize *E_B_*.

However, *E_B_* cannot completely determine the optimal tuning curves. This is because the problem is fundamentally under-constrained: because the network model is linear (**R = wr**), any transformation by an invertible matrix **A** such that **w** → **wA** and **r** → **A** ^−1^ **r** produces the same approximation and thus leaves the error unchanged. Therefore, additional conditions on **w** or **r** are required to make the solution unique. These conditions are crucial, in that they can lead to quite different results [6,40], but it is instructive to ignore them momentarily; this provides some intuition into the problem, as well as a lower bound on *E_B_*.

Before considering specific examples, it is important to discuss the key factors that will determine the solution. Intuitively, the tuning curves should match, as much as possible, the *N* target functions *F→*_α_. If all the functions are different, then clearly a lot of tuning curves will be needed for accurate approximation. In this case, “different” means “not highly correlated,” which in turn means that **Φ** will have large values along its diagonal (see below). On the other hand, if the functions *F→*_α_ are similar to each other, then very few tuning curves should suffice. Or, if more tuning curves are available, many of them can be used to cover specific regions where **Φ** varies more abruptly. Therefore, what matters when designing tuning curves is really the number of distinct functions that need to be approximated, as measured both by how big *N* is and how correlated the functions *F→*_α_ are.

The rest of this section formalizes this intuition and describes more precisely the dependence of the optimal tuning curves on **Φ**. The reader who wishes to skip the mathematical details may safely move on to the next section.

To better understand the effect of **Φ**, it is useful to decompose it using a special set of vectors (eigenvectors) and their corresponding coefficients (eigenvalues). The idea is to use the eigenvectors of **Φ** to construct the optimal tuning curves. Assuming that all stimuli are equally probable, the key property of **Φ** is that its *M* eigenvalues are all non-negative and add up to *M* (see Methods). When **Φ** results from averaging either just a few functions (≪*M*) or many functions with similar shapes, only a few eigenvalues are significantly larger than 0. Conversely, when the average involves many different functions, most eigenvalues are close to 1 and **Φ** is strongly diagonal.

Keeping these properties in mind, as well as the fact that *E_B_* varies between 0 and 1, now consider a single basis neuron. Assume that its tuning curve is proportional to an eigenvector of **Φ** with eigenvalue λ. In that case, *E_B_* depends on only two numbers, λ and a signal-to-noise ratio ρ that is equal to the mean response squared divided by the mean variance of the neuron. That is,

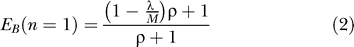
(see Methods). This expression leads to three important observations. (1) When the neuron's variance increases, ρ tends to 0 and the error tends to 1. Thus, as expected, higher noise always pushes the error toward its maximum. (2) The worst-case scenario is λ = 0. This produces the maximum error, regardless of the noise, and occurs when the tuning curve is completely different from (orthogonal to) all the target functions used to compute **Φ**. (3) For any signal-to-noise ratio, the lowest error occurs when λ is the largest eigenvalue of **Φ**, in which case the single tuning curve is equal to the so-called first principal component [41] of **Φ**. This one tuning curve may suffice to generate a very small error, if the noise is low and λ = λ^max^ ≈ *M*. But, on the other hand, if λ^max^ is small, the error will be large even if the tuning curve has the optimal shape and zero noise.

The efficacy of the single basis neuron thus depends on its variability, on the largest eigenvalue of **Φ**, and on the similarity between the tuning curve and the eigenvectors. An analogous result is obtained with more neurons, except that additional eigenvalues and eigenvectors become involved (see Methods). Specifically, with *n* basis neurons and no noise, the minimum error that can be achieved is

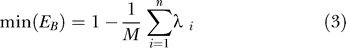
where λ_1_, … , λ*_n_* are the *n* largest eigenvalues of **Φ**. The key in this expression is that the sum involves *n* terms only. This is important because if **Φ** has just a few large eigenvalues, the sum of the *n* largest ones may approach *M* even if *n* ≪ *M*, so few noiseless tuning curves with the right shapes will suffice for representing accurately all the desired motor responses. This happens, for instance, when the motor responses are similar to each other, i.e., when they are highly correlated. Conversely, if many eigenvalues are close to 1, then **Φ** is strongly diagonal, and it is certain that a much larger number of sensory neurons will be needed even if noise is not a factor. Numerical results support these theoretical conclusions (see Protocol S1).

### Monotonic Versus Nonmonotonic Tuning Curves

Armed with a criterion that quantifies the accuracy of the sensory tuning curves and takes into account the statistics of the motor outputs, now we can ask: what sets of tuning curves are optimal when there is variability and when specific families of downstream functions are considered? To investigate this, each tuning curve was parameterized by four numbers, such that either monotonic or unimodal profiles with a large variety of shapes could be produced, and a numerical routine was used to find optimal parameter combinations that minimized *E_B_* (see Methods). By limiting the possible tuning-curve shapes, this procedure eliminated the ambiguity problem mentioned earlier.

**Figure 2:**
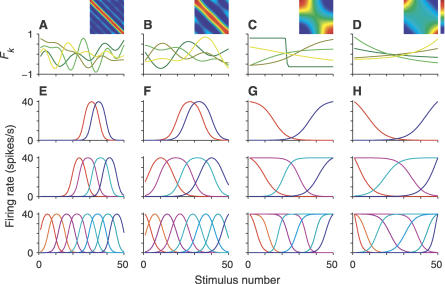
Optimal Tuning Curves for Four Classes of Downstream Functions (A) High-frequency oscillating functions. Each function *F→* was composed of eight sinusoids of random phase and amplitude. Four examples are shown. The inset depicts the correlation matrix obtained from 5000 functions. (B) Low-frequency oscillating functions. (C) Saturating monotonic functions. Each *F→* was an increasing or decreasing sigmoidal curve of random steepness and center point. (D) Nonsaturating monotonic functions. Each *F→* was an increasing or decreasing exponential curve with random steepness. (E–H) Optimal sets of two, four, and eight tuning curves for the classes in the corresponding columns. Shown responses minimized *E_B_* and were constrained to remain between 0 and 40 spikes/s.

Figure 2 illustrates the results of computer experiments in which optimal tuning curves were obtained numerically for four classes of downstream responses. Examples of functions within each class are shown on the top row, next to the corresponding **Φ** matrices (Figure 2A–2D). The graphs below show the sets of two, four, and eight tuning curves that minimized *E_B_* in each case. When the target functions are nonmonotonic (Figure 2A and 2B), the optimal tuning curves are themselves nonmonotonic (Figure 2E and 2F). Similarly, when the target functions are monotonic (Figure 2C and 2D), the optimal tuning curves are also monotonic (Figure 2G and 2H), even though the target classes comprise both increasing and decreasing functions. The detailed features of the optimal tuning curves clearly depend on the specifics of the target class. For instance, the number of peaks and troughs of the oscillating target functions affects the optimal width of the unimodal curves (compare Figure 2E and 2F). Most notably, however, because the noise properties and stimulus statistics remained constant, all the differences across columns are due to constraints that act downstream from the sensory neurons.

These results were highly robust with respect to various manipulations (Protocol S1). Increasing the noise, adding a power constraint, using nonuniform stimulus probabilities, or parameterizing the tuning curves differently did not alter the main finding: optimal tuning curves are predominantly monotonic or nonmonotonic depending on the type of downstream activity they are meant to evoke. Furthermore, manipulating the stimulus probabilities alone never gave rise to monotonic curves; for this, a monotonic trend in the downstream responses was necessary.

As mentioned in the Introduction, both unimodal and monotonic tuning curves are found in various parts of the brain, and this diversity has remained unexplained (see also the Discussion). The above results suggest that the two types of responses may arise not because of information-coding considerations but because of differences in the actions that various types of stimuli ultimately trigger. For instance, some stimulus parameters, such as the orientation of a bar, should lead to approximately the same sorts of movements regardless of the parameter's value. But other parameters or features, such as image contrast or sound intensity, have an obvious directionality, in that salient stimuli of high contrast or high intensity are more likely to lead to action. Thus, sensory neurons might respond in a qualitatively different way to features with and without such a behavioral bias, because that is the most effective way to generate the appropriate actions. The next two sections present two realistic situations where such motor asymmetries may arise.

### Mixed Tuning Curves for Binocular Disparity

**Figure 3:**
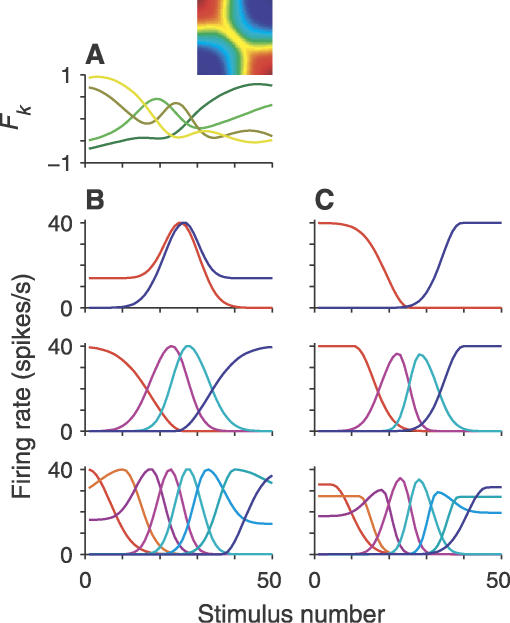
Optimal Tuning Curves for Downstream Functions That Have Both Peaked and Monotonic Components (A) Four examples of functions *F→* obtained by combining a localized oscillatory function (with a Gaussian envelope) and a saturating monotonic function. Such functions represent hypothetical motor responses to stimuli at various binocular disparities. The inset depicts the correlation matrix obtained from 5000 functions. (B) Optimal sets of two, four, and eight sensory tuning curves obtained with low noise. (C) As in (B), but with high noise and high power cost. In all plots, the *x*-axis represents binocular disparity.

Binocular disparity provides an interesting example of a signal that is likely associated with an intrinsic bias in behavior. To see the source of the asymmetry, consider what possible movements may be triggered by a visual stimulus at a given disparity. If a stimulus is seen near zero disparity (i.e., at the plane of fixation), many subsequent actions are possible, such as reaching, biting, fixating, etc. In contrast, if a relevant stimulus appears at a positive disparity (i.e., behind the plane of fixation), a diverging eye movement should typically follow, because that will bring the object onto the plane of fixation for more detailed examination. Conversely, converging eye movements should be seen more often after stimuli of negative disparity (i.e., in front of the plane of fixation). As a consequence, an oculomotor unit that is strongly activated at positive disparities should have a tendency to fire weakly at negative disparities, and vice versa. This rationale implies that for any relevant oculomotor cell, the responses at opposite ends of the disparity range should tend to be anticorrelated, and these should be approximately independent of the responses triggered near zero disparity. The downstream functions in Figure 3A are meant to capture this statistical regularity. They vary strongly in the middle of the stimulus range but have much more stereotyped values at the extremes.

Optimal tuning curves for this class of downstream functions are shown in Figure 3B and 3C. These curves have two novel features: they mix unimodal and monotonic profiles, and they include intermediate curves with a peak superimposed on a monotonic component. Recently, it has been shown that disparity tuning curves in area V4 have precisely these characteristics. The V4 population comprises a continuum of disparity tuning patterns that includes monotonic (the classic near and far cells), unimodal (the classic tuned cells), and intermediate cells [33].

### Widening Tuning Curves for Echo Delay

**Figure 4:**
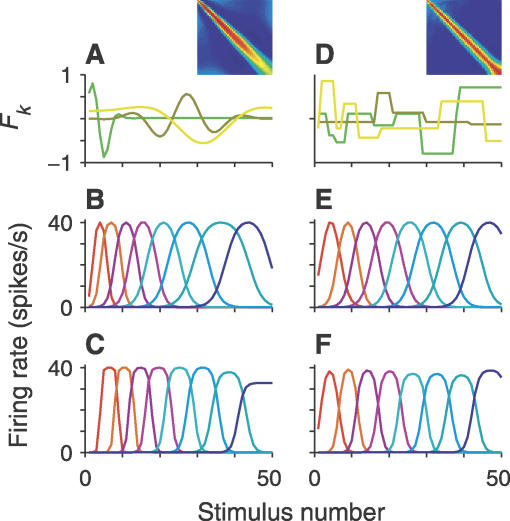
Optimal Tuning Curves for Downstream Functions That Vary More Rapidly near One End of the Stimulus Range (A) Three examples of continuous, oscillatory functions (with Gaussian envelopes) that oscillate at high frequency near stimulus 1 and at progressively lower frequency near stimulus 50. They represent hypothetical motor responses of bats as functions of echo delay or target distance. (B) Set of eight tuning curves that minimized *E_B_* given the correlation matrix in (A) and low noise. (C) As in (B), but with high noise and high power cost. (D) Three examples of discontinuous *F→* functions. Each one is a collection of constant segments placed randomly. Segment width increased linearly as a function of segment location on the *x*-axis. (E) Set of eight tuning curves that minimized *E_B_* given the correlation matrix in (D) and low noise. (F) As in (E), but with high noise and high power cost. In all plots, the *x*-axis represents echo delay.

The final example addresses the issue of tuning curve width. The downstream functions illustrated in Figure 4A are meant to capture a distinctive aspect of the behavior of bats, which locate prey by means of echolocation. In this case, consider a bat pursuing a moth. From far away, the bat can approach the moth by following its average path, smoothing out the moth's high-frequency maneuvers. At a close distance, however, the bat must turn at least as sharply as the moth itself in order to catch it, particularly in a cluttered environment [42,43]. Thus—this is the crucial assumption—when a bat flies toward a small target, its maneuvers must get faster as the target is approached. This postulate is translated into a statement about motor responses by generating functions that vary rapidly near stimulus 1 (corresponding to near targets, or short echo delays) and vary progressively more slowly at higher stimuli (corresponding to far targets, or long echo delays). Examples of such hypothetical motor responses are shown in Figure 4A. The optimal tuning curves for this case are nonmonotonic, as might have been expected, but most notably, their widths increase as functions of the preferred stimuli (Figure 4B). This effect is extremely robust. It was also observed when the tuning curves were parameterized differently and when high noise and high power cost were used (Figure 4C). Many auditory neurons of the bat's sonar system have this particular property. They are tuned to echo delay, and their tuning-curve widths vary linearly with the so-called “best delay” [44,45], which is the echo delay at which the peak response is elicited.

Again, note that the model generates this result based on a single statistical assumption about the motor responses, which is a progressive change in their absolute rate of variation along the echo-delay range. This is confirmed in Figure 4D, for which radically different downstream functions were generated. In Figure 4D, piecewise-constant functions were used, each composed of a variable number of segments that had random amplitudes and locations. The only structure was a correlation between segment length and segment location along the *x*-axis. It is this correlation that gives rise to the systematic change in tuning-curve width (Figure 4E and 4F).

A key question here, however, is whether curves of increasing width could also result from an uneven distribution of stimulus probabilities *s_k_* without assuming an asymmetry in the downstream functions. Technically the answer is yes—a progressive widening was obtained by using monotonically increasing stimulus probabilities together with the downstream functions in Figure 2A and 2B. But there were three severe problems with this purely sensory mechanism: (1) the effect required high noise; (2) it was much weaker, meaning that variations in width were small; and (3) most importantly, it placed the narrow tuning curves in the region of highly probable stimuli, which for the bat means that nearby targets must be encountered much more often than far away ones are. Therefore, the puzzling widening of sensory tuning curves documented in the bat may be explained more parsimoniously by assuming that flight control needs to be faster as the target gets closer.

### Other Tuning Curve Shapes

The parametric approach presented here allows a direct comparison between monotonic and peaked tuning curves. Would the results hold, however, if other shapes were allowed? To address this question, optimal tuning curves were recalculated using drastically different constraints. The basis responses were simply required to be positive and bounded, whereas the synaptic weights were constrained to be sparse. With sparse connectivity, each downstream function is approximated using only a subset of all the available basis neurons. The optimal tuning curves obtained with this method were much more variable, as expected given the absence of restrictions on their shapes, and they often had multiple peaks. However, an index measuring the monotonicity of the curves in each population was computed, and in terms of this index, the results were very similar to those obtained with parameterized curves: the monotonicity of the basis responses was determined by the monotonicity of the downstream functions, and conversely, strongly monotonic tuning curves could not be produced by manipulating the stimulus statistics alone. Details of these numerical experiments are discussed in Protocol S1.

## Discussion

Both unimodal- and monotonic-encoding populations of neurons are common and are maintained by different brain regions [16–20,26–31], including areas beyond the periphery where tuning curves seem to be actively synthesized [28,46]. Yet the factors that determine whether a specific neuronal population develops monotonic, unimodal, or mixed responses have remained a mystery. Computationally, unimodal curves are different from monotonic ones in two ways. First, they allow learning to be local, in the sense that changing the weight of a peaked curve affects the output function only over the range of the curve, not over the entire input space [36,38]. Second, it seems that representing multiple values simultaneously would be much easier with peaked curves, especially when the difference between coded values is large relative to the curve width. Although these differences still have unclear importance, they further illustrate the lack of theoretical justification for monotonic sensory responses. A possible solution to this enigma, however, is to consider the types of actions that various stimuli ultimately trigger.

### Maximizing Fisher Information Is Not Enough

The classical approach to sensory coding involves information maximization [25]. Thus, it would seem that some of the examples discussed above could be formulated in more familiar terms by requiring that more Fisher information [23,24], or equivalently, higher accuracy, be found in certain parts of the sensory space. For instance, in analogy with Figure 3A, what happens if much higher accuracy is needed in the middle of the stimulus range than at the edges? Could such conditions lead to monotonic tuning curves?

The answer is no. This is because a function specifying a desired relative accuracy at each point in the stimulus range is exactly equivalent to the set of coefficients *s_k_* that were used to represent stimulus probabilities. That is, *s_k_* can also be interpreted as the weight or importance of the error between driven and desired motor responses when stimulus *k* is presented (see Equation 6). For instance, when these coefficients had a Gaussian instead of a uniform profile, the results were entirely consistent with an increase in Fisher information at the middle of the range; more tuning curves were located near the middle, and those were narrower than the ones at the edges. These effects depended on the level of noise, as expected, and were rather subtle, but the key point is that such manipulations had no bearing on whether the optimal tuning curves were monotonic or not (see Protocol S1 for further results). Therefore, although information maximization is clearly important, the downstream functions in this model have a much stronger influence on the optimal tuning-curve shapes.

### Inputs, Outputs, and Optimality

Previous theoretical studies have attempted to explain the properties of sensory neurons based on two elements: an optimality assumption (efficient coding) and the statistics of their inputs; i.e., the statistics of natural images or natural sounds [3,5–9,13,14]. Conceptually, the approach here was not dissimilar. An optimality assumption, accurate function approximation, together with the statistics of motor responses were used to infer the shapes of sensory tuning curves. However, the present model works backwards in that it requires knowledge about downstream rather than upstream events (note, however, that stimulus statistics are still taken into account through the coefficients *s_k_* and through correlations with the downstream functions they evoke). Clearly, whereas measuring the statistics of natural images or sounds is straightforward, determining the statistics of motor activity associated with specific stimuli poses a challenge. However, assuming that such motor statistics have some structure, because of the animal's behavior, the results of the present model are straightforward: the shapes of the optimal sensory tuning curves should be adapted to that structure.

Two main conclusions follow from these results—a general one and a specific one. The general observation is that contrary to what is implicitly assumed in most studies, the optimality of sensory-triggered responses depends not only on their variability and on the statistics of stimuli but also on the downstream events driven by those responses [14]. If the downstream demands change, the responses considered optimal will change as well, at least as required by minimization of the performance measure used here, *E_B_*. One particular consequence of this is that the optimal width of peaked tuning curves is not uniquely determined by signal-to-noise considerations [23,25] (more on this below). This suggests that a comprehensive understanding of the firing properties of sensory neurons requires knowledge of the downstream impact of their responses.

In retrospect, this point may seem obvious. If the motor functions to be approximated are monotonic, so should be the tuning curves of upstream neurons that drive them. However, this idea has not been formally articulated before. Furthermore, previous explanations of key features of tuning curves—tuning-curve width, degree of overlap between curves, number of peaks, etc.—have always been based on arguments about coding efficiency. The simple model presented here indicates that such features may also generally depend on the motor actions performed by the animal. This, I believe, is a new insight, because it applies to neurons that are firmly considered as sensory.

The specific point is that monotonic and nonmonotonic curves are optimal under subtly different circumstances, which may depend on what can be termed a “behavioral bias.” This simply refers to an asymmetry in the relevant sensory stimulus. A bias exists when different parts of the stimulus range lead to different sets of possible actions, so that not all stimulus values are equal. The classes of downstream functions used here were meant to abstract this distinction in a simple way, and the results suggest that monotonic curves are efficient when there is such an asymmetry. Image contrast [30] and pressure on the skin [27] are good examples, because just on the basis of detection probability, high values are much more likely to lead to behavioral responses than low ones are. But in general, weaker or more restricted biases may lead to populations of neurons with both monotonic and peaked tuning curves, as seen experimentally [33–35].

### Model Predictions

If the model is correct, some variations in tuning properties across sensory populations should correspond to adaptations that enhance motor activity. Specifically in the case of arrays of Gaussian tuning curves [21–25], the model predicts that downstream motor responses should vary more rapidly in the stimulus range where the Gaussian curves are narrower. For instance, according to Figure 4, echolocating bats must compute motor functions that vary a lot around zero echo delay. Elegant experimental studies by Moss and collaborators are consistent with this interpretation. They show not only that the rate of turning of bats indeed increases as a target is approached [42,43], as was argued earlier, but also that their vocalizations speed up in several ways: (1) the rate at which sonar calls are emitted increases as the target gets near, (2) the duration of each call decreases, and (3) each frequency-modulated call consists of a sweep from a high to a low frequency, and the speed with which the frequencies are swept also increases. These three quantities vary by a factor of about three from the beginning to the end of a capture [43]. Furthermore, in the brown bat, microstimulation of the superior colliculus produces not only movements of the head and pinna but also sonar calls, where the number of evoked sonar pulses increases as a function of both the duration and the amplitude of the injected current [47]. These data strongly suggest that relevant motor neuron activity is indeed generally faster in the region where narrow tuning curves are found.

The model may also be useful for understanding sensory responses associated with escape or evasive behaviors in which the motor reaction should be faster as a potential threat approaches. This is a behavioral-bias scenario: if the likelihood or the speed of an evasive movement increases monotonically as a function of the proximity and speed of an incoming object, then one should expect the driving sensory neurons to have monotonic profiles. This indeed is reported to happen in several systems. For example, flying locusts make a characteristic dive when predator-sized stimuli are looming on one side. The key for triggering the glide is thought to be a single movement-detector unit, the so-called DCMD neuron, and this neuron fires with increasing frequency as the looming stimulus gets nearer [48]. Similar monotonic responses as functions of distance [49,50] and speed [19] have also been documented in other neurophysiological preparations where escape or collision avoidance is important. Even in monkeys, neurons that are sensitive to the distance of an object approaching the face seem to have monotonic dependencies on object distance (see Figure 4 in [51]). Likewise, cortical neurons that respond to optic flow, which are particularly useful for avoiding obstacles during locomotion [52], encode heading speed (i.e., the speed of one's own motion) in a predominantly monotonic way [53].

Perhaps the most counterintuitive consequence of the model is that when behavior does not require high accuracy, the sensory representation should be correspondingly coarse, even if in principle it could be made more precise. As illustrated in Figure 2B and 2F, when the motor response functions are broad, so should be the sensory tuning curves. An impressive data set collected by Heffner and colleagues supports this notion [54,55]. They have shown that sound localization capacity in mammals varies tremendously, with discrimination thresholds ranging from about 1° in humans and elephants to about 30° in mice and horses. These differences are not accounted for by variations in interaural distance, animal lifestyle, or environmental cues. Instead, “sound localization acuity in mammals appears to be a function of the precision required of the visual orienting response to sound” [54]. The argument is as follows. A primary purpose of auditory localization is to generate an orienting response, i.e., to bring the sound source into the fovea for detailed visual analysis. Consequently, species with small areas of best vision (e.g., human, elephant) need to generate highly precise movements, whereas species with large areas or streaks of best vision (e.g., mouse, horse) do not. Based on 24 mammalian species, the correlation between sound localization acuity and foveal width is 0.92. Crucially, however, the correlation with visual acuity itself is −0.31, so a purely sensory explanation again fails. According to classic sensory coding notions, species with high acuity must have tuning curves that are correspondingly narrower or less noisy. Therefore, in view of the behavioral data, large variations in the width of sound localization tuning curves are expected across species. These would be explained by motor constraints, as predicted by the theory. Similar interpretations should be possible in other systems, as long as sensory tuning curves can be directly related to clearly defined behaviors.

### Conclusion

The model developed here is based on an optimality criterion for neuronal tuning curves that takes into account both sensory (upstream) and motor (downstream) processes. This simple model is useful when sensory responses can be functionally related to specific behaviors, in which case it may explain some features of sensory representations that appear intriguing from the traditional perspective of sensory coding based on information maximization. In particular, this approach provides a theoretical rationale for the existence of monotonic tuning curves, which so far have lacked a plausible explanation, and it yields some insight into the apparently idiosyncratic varieties of sensory tuning curves observed across neurophysiological preparations.

### Materials and Methods

#### Numerical methods.

All calculations were performed using Matlab (The Mathworks, Natick, Massachusetts, United States). Results are shown for *n* between two and eight neurons and *M* = 50 stimuli. The mean response of neuron *i* as a function of stimulus *k*, where *k* = 1,…, 50, was parameterized as follows

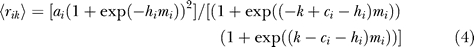
where *a_i_* is the amplitude of the curve for neuron *i, c_i_* is the center point, *h_i_* is the half width, and *m_i_* is a factor that determines the slope. This expression produces either unimodal curves, which may have positive or negative kurtosis, or monotonic curves, which may vary in steepness. The correlation matrix **C** was obtained by assuming that noise is independent across neurons, in which case *C_ij_* = 〈*r_ik_r_jk_*〉 = 〈*r_ik_*〉〈*r_jk_*〉 + δ*_ij_* 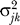 , where δ*_ij_* = 1 if *i* = *j* and is 0 otherwise. For each neuron *i*, the standard deviation of the noise during stimulus *k* was σ*_ik_* = α(*r*_max_/2 + 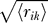 ), but other choices produced similar results. In the low- and high-noise conditions, *α* = 0.05 and 0.5, respectively.

Matrices **Φ** were produced directly by generating 5000 functions *F→*_α_ within a class and averaging the pairwise products *F*_α*k*_ *F_αl_*. The functions in each class were determined by small numbers of parameters. For example, for the saturating monotonic curves (Figure 2C), *F*_α*k*_ = *a* + *b*(1 + exp((*c* − *k*)/*d*))^−1^, where for each *α,* the center point *c* and slope factor *d* were chosen randomly within a range, and *a* and *b* were set to satisfy two normalization conditions. The first one was 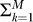 *s_k_F*_α*k*_ = 0 for all *α,* so the mean of each downstream function was set to 0. This was simply to shift the baseline of each *F→*_α_ and make the resulting **Φ** matrix easier to visualize in the plots; it had little or no effect on the optimal tuning curves. The second normalization condition was 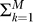 *s_k_*Φ*_kk_* = 1. It limited the amplitude of the downstream responses. Final values of Φ*_kl_* varied depending on the chosen class of functions, but never exceeded the range [−2.4, 4].

Given the terms *s_k_* and Φ*_kl_*, a routine searched for the combinations of parameters *a, h, c,* and *m* in Equation 4 that minimized *E_B_* (Equation 8). The minimization routine used the Nelder-Mead downhill simplex method [56]. A set of tuning curves was deemed optimal only after extensive testing and refining to ensure that the solution was near the global minimum. Additional constraints were included by adding suitable penalty terms to *E_B_*. For instance, to constrain the total power, a term proportional to 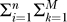 *s_k_* 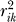 was added.

Tuning curves were also generated using a second parameterization (Figure 3 and Protocol S1). In this case, Equation 4 was substituted with a combination of two half-Gaussians with different widths and baselines but a common peak [33],

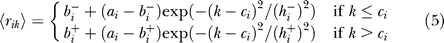
Here there are six free parameters per basis neuron: the center point *c_i_ ,* amplitude *a_i_ ,* left and right baseline levels 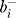 and 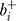 *,* and left and right widths 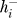 and 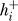 .

#### Derivation of *E_B_*.

The objective here is to derive an expression that quantifies how well the sensory tuning curves 〈**r**〉 approximate a desired set of downstream responses **F**. The standard procedure is to consider the average squared difference between driven and desired responses, i.e., the norm |**R** − **F**|. Because **R** = **wr**, this produces

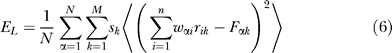
where the coefficient *s_k_* represents the probability of stimulus *k*, such that 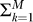 *s_k_* = 1, and the average indicated by angle brackets is over repeated presentations of a given stimulus. *E_L_* is the linear approximation error. This number quantifies how accurately the sensory (basis) neurons and associated synaptic weights are able to generate the desired motor activity downstream.

The next step is to obtain an expression for the error that no longer depends on the synaptic connections. To do this, the idea is to assume that the sensory tuning curves are known, and then find the set of synaptic weights **w** ^opt^ that minimize *E_L_*. These optimal weights are then substituted back into Equation 6, and the result is an expression for the mean square error that assumes that the synaptic connections are always the best possible ones. This is as follows.

First, find the optimal connections by calculating the partial derivatives of *E_L_* in Equation 6 with respect to *w_αi_* and equating the result to 0. This gives the optimal weights

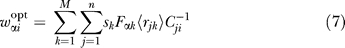
where **C** ^−1^ is the inverse of **C**, and *C_ij_* = 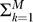 *s_k_*〈*r_ik_r_jk_*〉 is the correlation between sensory neurons *i* and *j*. The weights **w** ^opt^ generate linearly driven responses **R** that, on average, approximate the target motor responses as accurately as possible given the mean firing rates of the sensory neurons and the statistics of the stimuli.

Having minimized *E_L_* with respect to the connections, next, find out how big it is by substituting the optimal weights of Equation 7 back into Equation 6 and rearranging terms. Calling the result *E_B_*, this gives

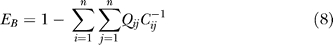
where

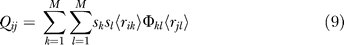
and Φ*_kl_* = (1/*N*) 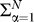 *F*_α*k*_ *F*_α*l*_, as mentioned in the main text. Thus, *E_B_* is a function of the first and second moments of the sensory responses, the stimulus probabilities, and the output correlations **Φ**.

Importantly, in the expression above, the following normalization condition was imposed: 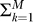 *s_k_*Φ*_kk_* = 1. This limits the amplitude of the downstream functions and bounds *E_B_* between 0 and 1. That the error cannot be negative follows directly from the definition of *E_L_* above. That it is bounded by 1 is not immediately obvious, but is a consequence of the fact that the eigenvalues of **Φ** are non-negative, and the normalization restricts their total sum. The next section gives more details.

#### Lower bounds on the approximation error.

In this section, the two analytic results discussed in the main text—Equations 2 and 3—are developed. The main expression derived below is, in fact, a slightly more general statement about the accuracy of the sensory tuning curves.

Here, a key simplifying assumption is that all stimulus probabilities are equal, so *s_k_* = 1/*M* for all *k*. As a consequence, the maximum eigenvalue of **Φ** satisfies 1 ≤ λ^max^ ≤ *M*. This important property is true for two reasons: first, because **Φ** results from the product of a matrix times its transpose, which guarantees that all its eigenvalues are non-negative; and second, because of the normalization condition on **Φ**, which in this case is

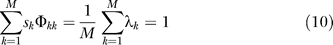
This condition makes the sum over all eigenvalues equal to *M*. Hence, λ^max^ is bounded between 1 and *M*.

To see how *E_B_* depends on the sensory responses, recall that their correlation matrix **C** is such that *C_ij_* = 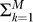 *s_k_*〈*r_ik_r_jk_*〉. Then, for a single basis neuron (*n* = 1) with mean response 〈*r_k_*〉, **C** becomes a scalar *C* = *rİ* ^2^ + σİ^2^, where

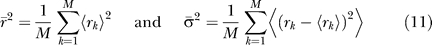
Also, if the tuning curve is an eigenvector of **Φ** with eigenvalue λ, then 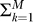 Φ*_kl_*〈*r_l_*〉 = λ〈*r_k_*〉, by definition, and Equation 9 gives *Q* = λ* rİ* ^2^/*M*. Substituting into Equation 8 and defining ρ = *rİ* ^2^/ σİ^2^ leads to Equation 2, which is the approximation error for a single neuron.

With more neurons, it is possible to derive a lower bound on the error that is more general than Equation 3. First, assume that *s_k_* = 1/*M* and that the noise has equal magnitude and is uncorrelated across neurons, such that

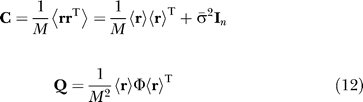
where **I** *_n_* is the *n × n* identity matrix and σİ^2^, the variance averaged over stimuli, is the same for all neurons. To proceed, consider the singular value decomposition (SVD) of the matrix of mean responses, 〈**r**〉 = **uSV** ^T^, where **u** is an *n × n* unitary matrix, **V** is an *M × M* unitary matrix, and **S** is an n *× M* matrix with *n* singular values along the diagonal and zeros elsewhere [56]. This is assuming that the *n* tuning curves are independent; if not, then the number of nonzero elements of **S** will equal the number of independent curves (the rank of 〈**r**〉). The SVD is a generalization to rectangular matrices of the classic eigenvalue decomposition. Substituting **C** and **Q** into Equation 8 and using the SVD representation of 〈**r**〉 leads to

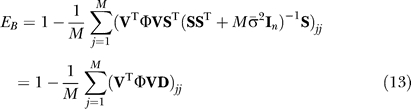
The first equality results from the defining property of unitary matrices, such that **uu** ^T^ = **I** *_n_* and **VV** ^T^ = **I** *_M_*. The second equality results from grouping into **D** all the terms involving **S**. The matrix **D** turns out to be *M × M* and diagonal, with entries *D_i_* = 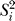 /(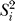 + *M* σİ^2^), where a single index is used to indicate relevant elements in diagonal matrices. Note, however, that only the first *n* diagonal elements are nonzero, because **S** itself only has at most *n* nonzero singular values along the diagonal (recall that **S** is diagonal but rectangular, *n × M*). The lower bound on the expression above thus involves a sum of only *n* terms; the bound is

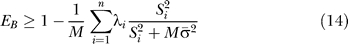
where λ_1_, … , λ*_n_* are the *n* largest eigenvalues of **Φ**.

To see this, first write **Φ** in terms of its eigenvalue decomposition, so that **V** ^T^ **ΦV** = **V** ^T^ **EΛE** ^T^ **V**, where **E** is the matrix of (right) eigenvectors of **Φ**. Suppose that the eigenvalues **Λ** are sorted in decreasing order, so that λ_1_ is the largest. Now note that the diagonal elements of the matrix **V** ^T^ **ΦV** depend on the match between **V** and **E**. In particular, the best possible match occurs when **V** is identical to **E**; then **V** ^T^ **EΛE** ^T^ **V = Λ**, and the equality in Equation 14 follows directly from Equation 13. This means that equality is obtained when the basis tuning curves are constructed using the eigenvectors of **Φ** sorted in decreasing order (i.e., **V** is equal to **E**). In contrast, if, for example, **V** has the same columns as **E** but sorted in the reverse order, then the resulting sum is similar to that in Equation 14 except that it involves the *n* smallest eigenvalues. That *E_B_* varies between 0 and 1 follows from Equation 10.

Equation 14 is the main analytic result and provides important intuitions about the mean basis responses, or sensory tuning curves, 〈**r**〉. These are as follows.

With *n* = 1, the result is Equation 2 written as an inequality, with the signal-to-noise ratio equal to *S* ^2^/(*M* σİ^2^). Also, Equation 3 is obtained when σİ^2^ = 0.

Noise always increases the error, because σİ^2^ effectively decreases every eigenvalue in Equation 14. However, noise partially determines the optimal shapes of the tuning curves. For example, if λ_1_ > λ_2_ but *S*_2_ >> *S*_1_ *,* then the second eigenvector should take precedence over the first, because its signal-to-noise ratio will be much higher. In other words, in this case, the first column in **V** should contain the second eigenvector of **Φ**. Thus, noise also determines which eigenvectors should be chosen in what order, and therefore the optimal shapes of the basis responses.

Because of the last point, noise helps solve the ambiguity discussed earlier—that the set of basis responses is determined up to an invertible transformation. However, it does not entirely solve the problem, and this is why. When σİ = 0, both matrices **u** and **S** are absent from Equation 14. Therefore, they are arbitrary; they do not affect the error (as long as they are unitary and diagonal, as required). In contrast, with noise, there is a criterion for setting the *S_i_* values, so only **u** remains arbitrary. Thus, without noise, 〈**r**〉 is ambiguous up to an invertible transformation, whereas with noise, it is ambiguous up to a unitary transformation.

In addition, it is important to mention that this ambiguity remains when the stimulus probabilities are not uniform. When arbitrary probability values *s_k_* are included in the calculation of Equation 13, the resulting expression for the error still does not depend on **u**. Therefore, manipulating the stimulus statistics does not solve this problem.

Finally, to minimize *E_B_* in the presence of noise it is best to increase 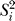 as much as possible. However, the total power in the mean responses is 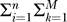 〈*r_ik_*〉^2^ = 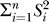 . Therefore, with noise, additional constraints that effectively limit the power are necessary to obtain optimal responses of finite amplitude.

## Supporting Information

Protocol S1 Combined Supporting Information (154 KB PDF)
