# Supplementary material for: How Behavioral Constraints May Determine Optimal Sensory Representations
Source: PLoS Biol. 2006 Nov 28;4(12):e387. doi: 10.1371/journal.pbio.0040387 (PMC1661681; doi:10.1371/journal.pbio.0040387)
Supplement: Supplementary file 1 [file pbio.0040387.sd001.pdf]

Supporting Information

Main text: Salinas E (2006) How behavioral constraints may determine optimal sensory representations. PLoS Biology.

Parameter Manipulations

Figure S1 shows additional results in which the same four classes of target functions shown in Figure 2 were used but other aspects of the computer experiments were varied. In Figure S1A, the noise of the basis responses was much higher. This caused all tuning curves to rise and fall more steeply than with low noise. This makes sense because, to increase the signal-to-noise ratio as much as possible, higher firing rates are necessary. In Figure S1B, a term penalizing the total power of the responses was added to the error (see Methods), which amounts to putting an energy cost on the neural activity. This tends to favor unimodal neurons, which typically have smaller mean firing rates across all stimuli than monotonic ones. In this case, unimodal curves reduced their amplitudes and monotonic curves shifted toward the edges. Also, a monotonic curve was exchanged for a unimodal one. Such exchanges occur only when adding a neuron results in a large power penalty, larger than the corresponding decrease in E_B. In the examples shown,

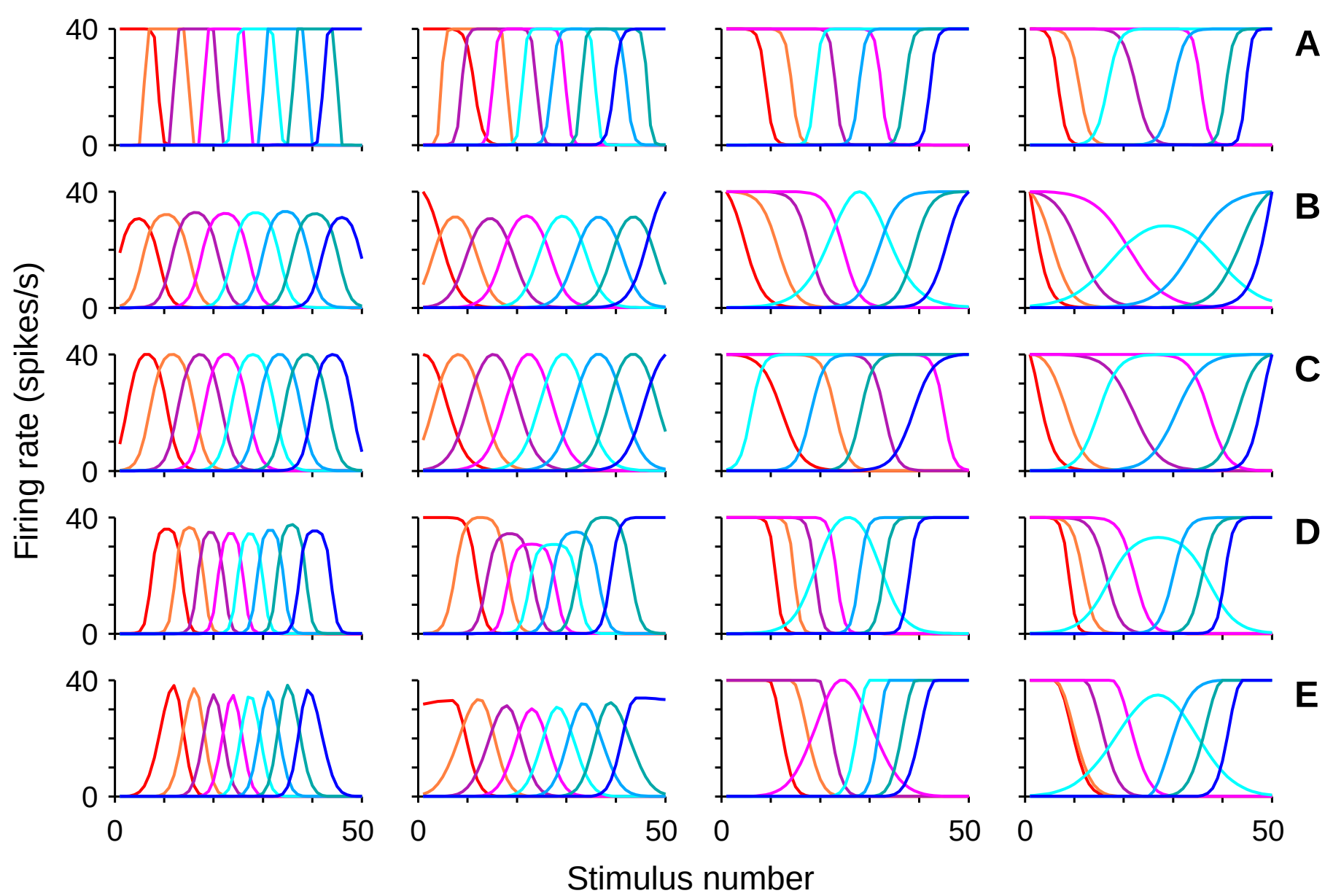

Figure S1. Sets of 8 optimal tuning curves obtained under various conditions. Columns correspond to the same classes of downstream functions as in Figure 2A–D. Numerical experiments were as in Figure 2, except for changes stated explicitly. (A) High noise. The SD of the firing rates was ten times as high as in Figure 2. (B) High power cost. A penalty term proportional to the sum of the squared responses was added to E_B. (C) Unequal stimulus probabilities. The probability profile (s_k as a function of k) was Gaussian with an SD of 7. The stimulus in the middle of the range was about ten times more frequent than those at the edges. (D) Combined conditions. The three previous manipulations were applied simultaneously. (E) As in (D), but with the tuning curves parameterized in an alternative way (Equation 5).

E_B was already very low with 7 neurons, so the advantage in accuracy of an additional monotonic curve was too small relative to its high mean rate. In Figure S1C, the stimulus probabilities were not uniform. Instead, they had a Gaussian profile centered on the middle stimulus (see Figure S4F). This caused the center points of the tuning curves to shift very slightly toward the high-frequency stimuli. The effect, however, was much stronger in conjunction with the other manipulations, as shown in Figure S1D. Here, all three changes — high noise, high power cost, and unequal stimulus probabilities — were applied simultaneously. The tuning curve locations varied strongly but their shapes remained qualitatively the same. Finally, to investigate whether the results depended on the specific tuning curve parameterization that was chosen, all numerical experiments were repeated with a second parameterization that allowed tuning curve profiles intermediate between unimodal and monotonic; this was the same parameterization used by Hinkle and Connor [33] (see Methods and Figure 3B). In all cases, the results were similar to those obtained earlier. Figure S1E shows an example in which the same conditions of Figure S1D were used. Although the individual shapes are slightly different, as expected, all curves are either unimodal or monotonic — no intermediate curves were generated.

In summary, then, the results are not overly sensitive to noise, power constraints, stimulus probabilities, or the specific way in which tuning curves are defined mathematically.

An Alternative Set of Constraints

A different approach was also explored in which Equation 6 was directly minimized with respect to $\mathbf{w}$ and $\langle \mathbf{r} \rangle$ using a modified gradient descent algorithm. In this case, both $\mathbf{w}$ and $\langle \mathbf{r} \rangle$ were updated iteratively. Tuning curves were constrained to vary between 0 and 40, which was enforced by renormalizing all modified curves after every update. In addition, the synaptic connections were forced to be sparse by adding to Equation 6 a penalty term proportional to

$$\sum_{\alpha}^{N} \sum_{i,j \neq i}^{n} |w_{\alpha i}\, w_{\alpha j}| + \sum_{i}^{n} \sum_{\alpha,\beta \neq \alpha}^{N} |w_{\alpha i}\, w_{\beta i}| \,. \tag{15}$$

Here, the first term represents the synaptic redundancy across rows of $\mathbf{w}$, whereas the second term represents the redundancy across columns. For instance, the penalty term for row α is $\sum_{i,j\neq i}^{n} |w_{\alpha i}\, w_{\alpha j}|$. This expression is minimized when only one of the connections to downstream neuron α is not zero, in which case the actual value of the non-zero weight does not matter. This type of penalty thus tends to produce many synaptic weights equal to zero. When the number of basis neurons is equal to the number of downstream neurons, this constraint assigns one basis neuron to one downstream neuron (if no other restrictions are imposed), making the connectivity matrix equivalent to the unit matrix. Thus, an intuitive interpretation of this constraint is "construct each downstream response using as few basis neurons as possible".

The results that follow were obtained by minimizing E_L (Equation 6) plus the expression above multiplied by a constant that determined the penalty strength. To use as few restrictions as possible, the motor responses $\mathbf{F}$ and the sensory tuning curves were made to vary between 0 and 40; no other normalization conditions were invoked.

Figure S2 illustrates the effect of the sparse connectivity constraint. Here, $\mathbf{F}$ consisted of increasing and decreasing sigmoidal functions, as in Figure 2C. Without the sparseness

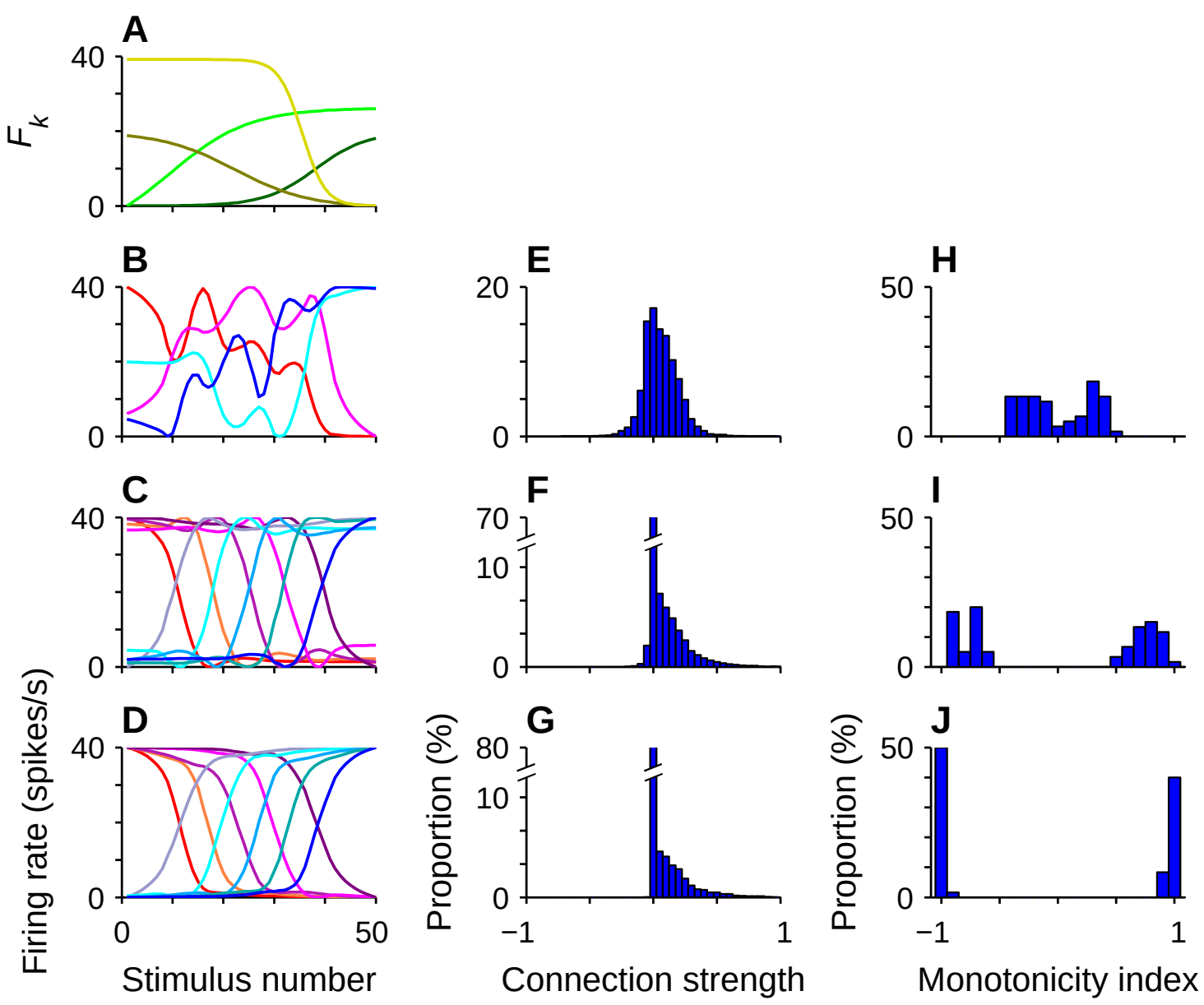

Figure S2. Optimal tuning with sparse connectivity. Results are shown for $\mathbf{F}$ with 2000 downstream functions and sets of 10 basis functions. (A) Four examples of desired downstream responses. (B) Four examples of optimal tuning curves obtained with no connectivity constraint (penalty strength was zero). (C, D) Optimal tuning curves obtained with penalty strengths of 0.002 and 0.02, respectively. (E–G) Distributions of synaptic weight values for penalty strengths of 0, 0.002 and 0.02, respectively. (H–J) Distributions of monotonicity indices for penalty strengths of 0, 0.002 and 0.02, respectively.

constraint, the distribution of optimal connections that results is approximately normal (Figure S2E), and the corresponding optimal basis functions have multiple peaks and no particular structure (Figure S2B). This is because, as mentioned in the main text, without additional constraints on $\mathbf{w}$ or $\langle \mathbf{r} \rangle$ the minimization of E_L is an ill-posed problem; there is no unique solution. In contrast, with the sparseness constraint in place, a large fraction of the synaptic connections become zero (Figure S2F), and the optimal basis responses are uniformly spaced and almost perfectly monotonic (Figure S2C). These effects increase with the strength of the penalty term (Figure S2D and S2G).

To quantify the monotonicity of the resulting basis responses, the derivative of each tuning curve was computed numerically, and a monotonicity index for each curve was calculated from it. Defining $d_{ik} = \langle r_{ik+1} \rangle - \langle r_{ik} \rangle$, the monotonicity index for cell i is

$$MI_i = \frac{\sum_k d_{ik}}{\sum_l |d_{il}|} \,. \tag{16}$$

This number goes from -1 for a monotonically decreasing curve to $+1$ for a monotonically increasing curve, with values near 0 indicating about equal numbers of increasing and decreasing steps. Figure S2H–J shows how the distribution of monotonicity indices changes as the strength of the sparseness constraint is increased. To construct the histograms, optimal sets of ten tuning curves were obtained six times with different initial conditions, producing slightly different sets. The 60 indices were then pooled to produce the shown distributions. The values are clustered near 0 when the basis tuning curves oscillate and reach ± 1 when they are monotonic.

The results in Figure S2 show that the sparse-connectivity constraint is effective at disambiguating the shapes of the optimal tuning curves and may lead to monotonic profiles. Importantly, however, monotonic curves are produced only when the target downstream functions are themselves monotonic or have monotonic components. This is shown in Figure S3. When the desired downstream responses are oscillatory and lack any direc-

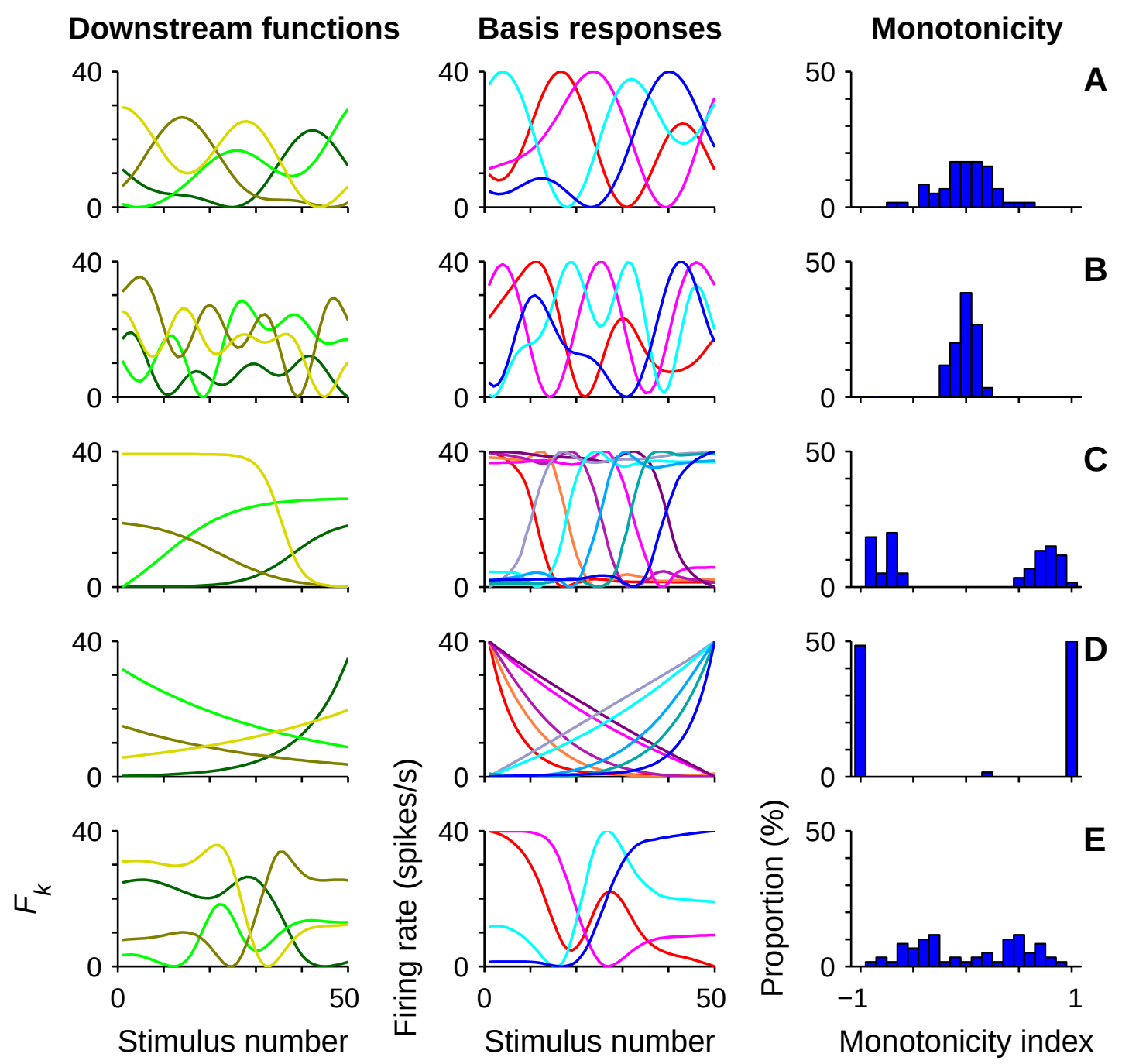

Figure S3. Optimal tuning curves for five classes of motor functions. Results are as in Figure S2, with a penalty strength of 0.002. Each row shows results for one class of downstream functions. Each panel in the left column shows four representative examples of functions of a given type; the middle column shows the resulting optimal basis responses; the right column shows their distributions of monotonicity indices. (A) Low-frequency oscillating functions. For clarity, only four of ten tuning curves are shown. (B) As in (A), but for high-frequency oscillating functions. (C) Increasing and decreasing sigmoidal curves (as in Figure S2C). (D) Exponential functions. (E) Motor functions that oscillate but have an increasing or decreasing trend (as in Figure 3).

tional bias, the resulting basis functions are themselves oscillatory, and their monotonicity indices are clustered around zero (Figure. S3A and S3B). Most interestingly, for the downstream functions used in Figure 3, which model hypothetical reactions to binocular disparity signals, the result is again an intermediate representation where the optimal tuning curves have various degrees of monotonicity and thus a widely-spread distribution of monotonicity indices (Figure S3E).

Influence of Stimulus Statistics

One particular question that was thoroughly investigated using this alternative set of constraints was whether the statistics of the stimuli could affect the tendency of the basis neurons to develop monotonic responses. The left-most column in Figure S4 shows four probability profiles that were tested. The flat one at the top is the standard, where all stimuli are equally probable ($s_k = 1/M$). In the other three, some stimuli are a lot more frequent than others. Sets of ten optimal basis responses were obtained with each profile when the goal was to approximate either oscillatory or monotonic downstream functions (Figure S4A and S4B). Figure S4 shows results obtained using a high level of noise, which tended to enhance the effects of the probabilities, as observed earlier with the parametric approach (Figure S2C and S2D). As can be seen by comparing the resulting tuning curves in different rows, the basis responses deemed optimal did adapt according to the probabilities. In particular, with non-monotonic downstream responses the variations in tuning curve amplitude were a lot smaller in the regions where stimuli had low probabilities. And with monotonic downstream responses it was the spread of the tuning curves that varied appreciably. However, in both cases the degree of monotonicity was barely affected by the stimulus probabilities. The two columns under Figure S4A show that when the target

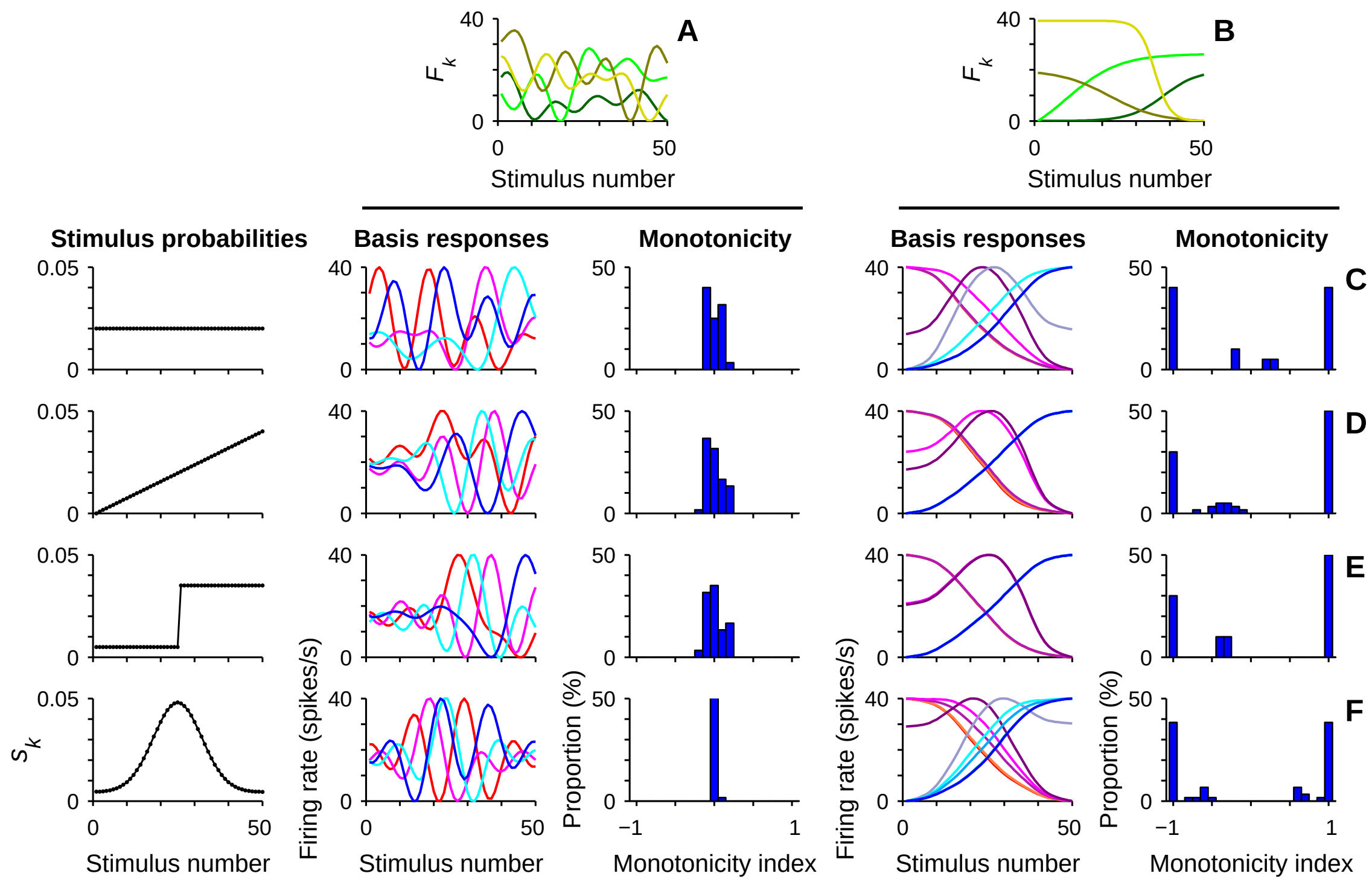

Figure S4. Effect of stimulus statistics. Sets of 10 optimal basis responses were obtained for oscillatory (A) and monotonic (B) downstream functions using 4 stimulus probability profiles (left-most column). A profile corresponds to the probability s_k as a function of stimulus k. Each condition was repeated 6 times to obtain 60 monotonicity indices. Optimal sensory responses and histograms of monotonicity indices are shown for each condition, as indicated in the respective columns. (C) Uniform profile. All stimuli were equally frequent. (D) Linear profile. Stimulus probability increased steadily. (E) Step profile. Stimulus probability changed abruptly at stimulus 25. (F) Gaussian profile. Stimuli in the middle of the range were much more probable than at the edges. All results are as in Figure S3 (penalty strength of 0.002), except that high noise was used ($\alpha = 0.5$). Monotonicity indices were highly insensitive to stimulus probabilities.

functions are non-monotonic the optimal tuning curves should also be non-monotonic, regardless of the probabilities s_k. Similarly, the two columns under Figure S4B indicate that when the target functions are monotonic the optimal tuning curves should also be predominantly increasing or decreasing, and the probabilities s_k barely make a difference in this regard. Therefore, it seems that monotonic tuning curves cannot be generated simply by manipulating the stimulus statistics. And furthermore, if monotonic curves are optimal because of downstream requirements, it seems that the statistics of the stimuli cannot override this trend.

Match between Analytic and Numerical Results

When a specific correlation matrix $\mathbf{\Phi}$ is chosen, Equations 3 and 14 make a prediction about how the mean square error should decrease as a function of the number of tuning curves (basis functions) used for approximating the corresponding downstream responses. Verifying that the numerical results match these expressions is important, first, to check that the minimization routines used to find the optimal basis functions are working

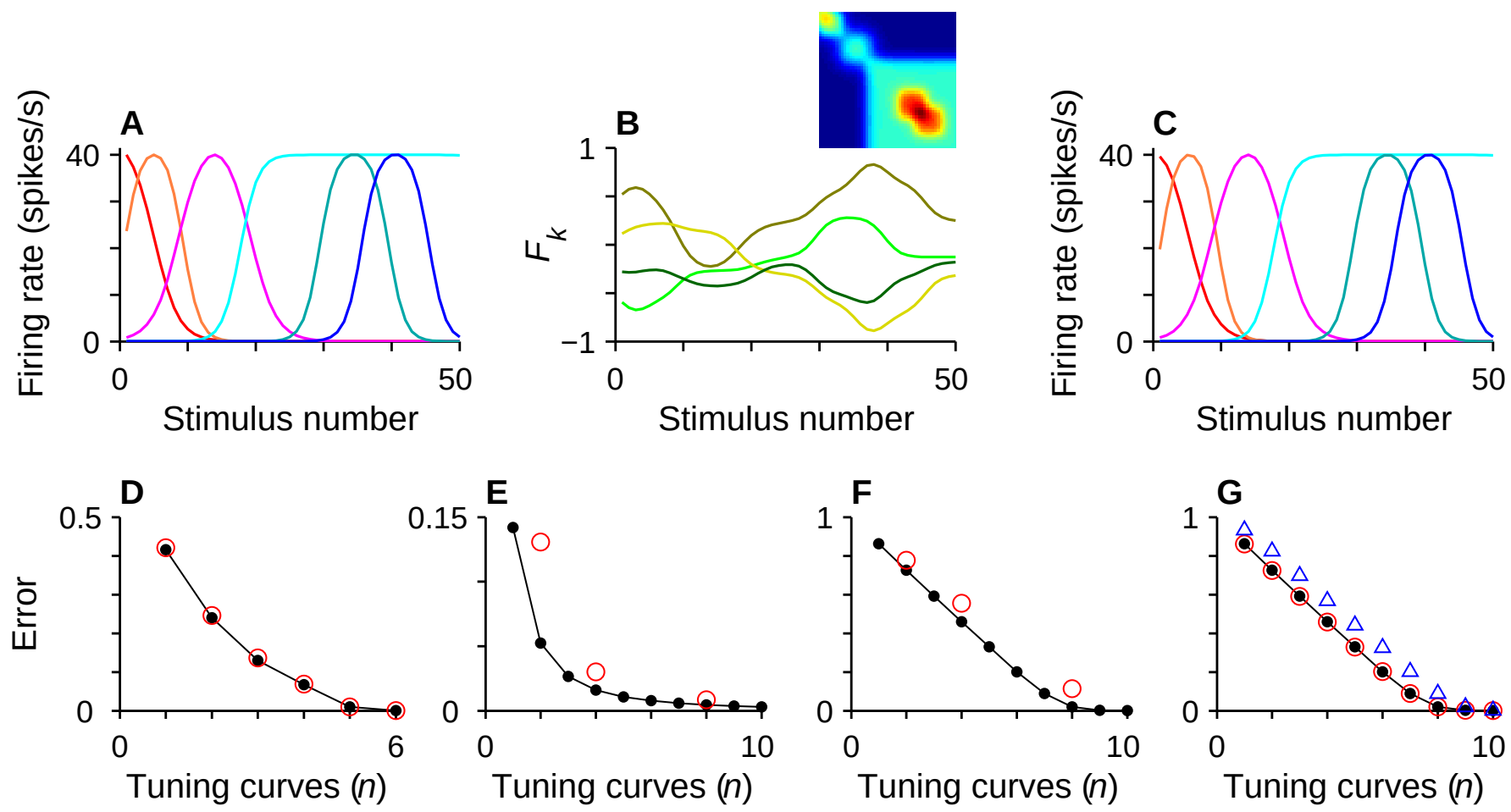

Figure S5. Mean squared error in the approximation versus theoretical minimum. (A) Set of functions used to construct the downstream responses shown in (B), which served as controls. Each of 5000 downstream curves was generated as a linear combination of the six responses shown in (A), with random coefficients. The resulting correlation matrix $\mathbf{\Phi}$ is in the inset. (C) Set of six optimal tuning curves recovered by the minimization routine. The curves shown minimized E_B given the control matrix $\mathbf{\Phi}$ in panel (B) and zero noise. (D) Mean squared error E_B as a function of the number of optimal tuning curves, n, when the $\mathbf{\Phi}$ matrix in (B) was used. Black dots are the minimum errors calculated from Equation 3. Red circles are the E_B values obtained (from Equation 8) with the indicated number of basis tuning curves. (E) As in (D), but when the $\mathbf{\Phi}$ matrix for sigmoidal functions shown in Figure 2C was used. Red circles correspond to the sets of 2, 4 and 8 curves shown in Figure 2G. (F) As in (E), but when the $\mathbf{\Phi}$ matrix for oscillatory functions shown in Figure 2A was used. Red circles correspond to the sets of 2, 4 and 8 curves shown in Figure 2E. (G) Optimal tuning curves for the oscillatory functions shown in Figure 2A were computed using the alternative method with no restriction on the synaptic weights (sparseness not enforced). Red circles are for optimal tuning curves only constrained to have a maximum of 1. Blue triangles are for curves constrained to have a maximum of 1 and a minimum of 0. Black dots are the same as in (F). No noise was used in (D) and (G).

properly, and second, to investigate whether few basis functions may indeed be sufficient to approximate accurately a large number of downstream responses, as implied by the analysis.

To address this, a special set of downstream responses was generated which, by construction, could be approximated exactly by the family of parameterized curves — single-peak or monotonic — used in Figure 2. This was done in three steps. (1) An arbitrary set of six peaked and monotonic curves was generated using the regular parameterization of Equation 4; these six curves are shown in Figure S5A. (2) A set of control downstream functions were constructed by adding the six curves in random proportions. Thus, each downstream response was equal to a linear combination of the six curves with coefficients drawn randomly from $[-1, 1]$. Four examples of such downstream functions are shown in Figure S5B, along with the resulting $\mathbf{\Phi}$ correlation matrix. (3) This control $\mathbf{\Phi}$ matrix was input to the minimization routine, which searched for the sets of 1, 2,. . ., 6 optimal tuning curves that minimized E_B. Note that the minimization routine had no information about the downstream functions other than $\mathbf{\Phi}$. The set of six optimal tuning curves ($n = 6$) found

by the routine is shown in Figure S5C. They are almost identical to the original set used to construct the control $\mathbf{\Phi}$. The mean square error as a function of the number of optimal tuning curves used in the approximation (n) is shown in Figure S5D. The actual error values (red circles) are superimposed on the minimum values (black dots) expected from Equation 3. The numbers are very close, showing that the minimization routine indeed found the best possible basis functions. They are not exactly equal, particularly for the first three points, because the shapes of, say, the best two basis functions ($n = 2$) cannot be perfectly fit by the parameterization of Equation 4. The error, however, is virtually zero for $n = 6$, where by construction the parameterized curves can indeed reproduce the best basis functions exactly.

What happens with other correlation matrices $\mathbf{\Phi}$? An example is shown in Figure S5E, which plots the mean square error (red circles) obtained in approximating the sigmoidal motor responses of Figure 2C with the sets of 2, 4 and 8 tuning curves shown in Figure 2G. Black dots are again the minimum values obtained from Equation 3, except that now the eigenvalues of the $\mathbf{\Phi}$ matrix in Figure 2C were used. Note that the errors are generally small; just two basis functions ($n = 2$) capture more than 85% of the variance of the downstream responses. This is because in this case all the motor responses are rather similar to each other. In contrast, Figure S5F shows an analogous plot that was generated using the $\mathbf{\Phi}$ matrix in Figure 2A, which resulted from oscillatory motor responses. In this case, both the minimum expected error (black dots) and the actual approximation error obtained with 2, 4 or 8 tuning curves (red circles) start much higher. The difference between them, however, is again small.

In Figure S5E and S5F the red and black dots differ for two reasons, first, because the noise was not zero, and any noise increases the approximation error (Equation 14), and second, because the parameterized curves could not match exactly the shapes of the optimal tuning curves. In all the examples studied, the error due to this mismatch in shape was equivalent to about one tuning curve. Thus, the error obtained with eight tuning curves was approximately equal to the minimum error calculated with seven eigenvalues, and so on. This is best illustrated in Figure S5G, which was constructed as follows. The black dots are the minimum errors for approximating the oscillatory motor responses of Figure 2A; they are the same as in Figure S5F. The other data points are the errors obtained with n tuning curves found using the alternative method mentioned in the previous section. That is, gradient descent was used to find sets of n tuning curves and connection weights that minimized E_L. Here, no noise and no restrictions whatsoever were placed on the synaptic weights. The red circles were obtained when the only constraint on the tuning curves was that their maximum had to be equal to 1. This did not limit the range of tuning curve values (which could be negative), nor the shape of the profiles, so the approximation errors were virtually equal to the theoretical minima. On the other hand, the blue triangles were obtained with the same method, but when the tuning curves were constrained to have a minimum of 0 and a maximum of 1. This limited their range of values and caused a small additional error. However, with more than 4 or 5 tuning curves, the approximation error with n tuning curves was almost exactly equal to the minimum (theoretical) error with $n - 1$ curves.

In conclusion, the families of downstream responses studied here can indeed be described by a few eigenvalues and eigenvectors. As a consequence, few tuning curves may

be enough to approximate a large variety of motor responses even when the range of tuning curve shapes is limited.

Note on the Number of Motor Responses

The $\mathbf{\Phi}$ matrices shown here were produced by averaging 5000 downstream functions. Such large number was used so that, for a given class of functions, the corresponding $\mathbf{\Phi}$ matrices would change little from run to run. With this number, two matrices generated with different groups of 5000 functions from the same class were virtually indistinguishable, and so were their eigenvalues. This was convenient for eliminating a potential source of variability across runs, and this in turn was important to ensure that the results were repeatable and that the solution was not a local minimum of E_B. However, adequate (smooth) $\mathbf{\Phi}$ matrices could also be obtained with 200 downstream functions or less, depending on their type.

The condition that there should be more motor neurons than sensory neurons ($n < N$) is crucial for the present framework. It is a reasonable assumption if one considers that a given sensory stimulus, say, a phone ringing, may trigger a variety of motor actions, such as answering the phone, ignoring it, listening to the answering machine to find out what the caller wants, etc. So, combined with the different contexts in which a stimulus may appear, the number of associated motor responses may be very large.
